# Myricetin suppresses traumatic brain injury-induced inflammatory response via EGFR/AKT/STAT pathway

**DOI:** 10.1038/s41598-023-50144-x

**Published:** 2023-12-20

**Authors:** Chenxing Wang, Siguang Ouyang, Xingjia Zhu, Yi Jiang, Zhichao Lu, Peipei Gong

**Affiliations:** 1 Department of Neurosurgery, Affiliated Hospital of Nantong University, Medical School of Nantong University, Nantong, 226001 Jiangsu China; 2 Research Center of Clinical Medicine, Affiliated Hospital of Nantong University, Nantong, 226001 Jiangsu China

**Keywords:** Biochemistry, Neuroscience, Drug development

## Abstract

Traumatic brain injury (TBI) is a common disease in neurosurgery with a high fatality and disability rate which imposes a huge burden on society and patient's family. Inhibition of neuroinflammation caused by microglia activation is a reasonable strategy to promote neurological recovery after TBI. Myricetin is a natural flavonoid that has shown good therapeutic effects in a variety of neurological disease models, but its therapeutic effect on TBI is not clear. We demonstrated that intraperitoneal injection of appropriate doses of myricetin significantly improved recovery of neurological function after TBI in Sprague Dawley rats and inhibited excessive inflammatory responses around the lesion site. Myricetin dramatically reduced the expression of toxic microglia markers generated by TBI and LPS, according to the outcomes of in vivo and in vitro tests. In particular, the expression of inducible nitric oxide synthase, cyclooxygenase 2, and some pro-inflammatory cytokines was reduced, which protected learning and memory functions in TBI rats. Through network pharmacological analysis, we found that myricetin may inhibit microglia hyperactivation through the EGFR-AKT/STAT pathway. These findings imply that myricetin is a promising treatment option for the management of neuroinflammation following TBI.

## Introduction

Traumatic brain injury (TBI) is a major cause of morbidity and mortality worldwide. However, the treatment of TBI is costly, and patient outcomes are generally unfavorable^1,2^. Neuroinflammation after CNS injury is a double-edged sword. On the one hand, neuroinflammation performs the functions of pathogen removal, tissue repair and neuroprotection. On the other hand, excessive neuroinflammation exacerbates nerve damage, impairs cognitive function, and induces anxiety and depression. The intensity and duration of inflammation largely determine whether immune signals are supportive or destructive to the CNS. Although the exact mechanisms underlying TBI are not fully understood, emerging evidence supports the idea that heightened post-injury neuroinflammation plays a significant role in its pathophysiology^3,4^.

Microglia play a critical role in the immune response of the central nervous system (CNS) and the maintenance of brain homeostasis, safeguarding the CNS against various harmful factors, such as infection and injury^5,6^. Activation of microglia is one of the initial signs of neuroinflammation, triggering the release of numerous chemokines, upregulation of cyclooxygenase (COX-2) and inducible nitric oxide synthase (iNOS), and an increase in the levels of prostaglandin E2 (PGE2). Furthermore, the levels of expression of pro-inflammatory cytokines such as interleukin-1β (IL-1β), interleukin-6 (IL-6), and tumor necrosis factor-α (TNF-α) increase rapidly. The inflammatory substances produced by the activated microglia switch on the nuclear factor (NF)-κB, protein kinase B (AKT), and signal transducer and activator of transcription (STAT) signaling pathways^7^.

Sustained activation of these pathways leads to excessive production of cytotoxic mediators and pro-inflammatory cytokines by microglia. This excessive neuroinflammation in the TBI lesion area leads to the deterioration of neurological function. Therefore, reducing neuroinflammation caused by microglia activation appears a potential approach to prevent psychiatric sequelae and increase neurological functional recovery after TBI^8^.

Myricetin, a naturally occurring flavonoid found in red wine, fruits, berries, tea, and medicinal herbs, has similar physicochemical properties to morphine due to its structural similarity^9^. Furthermore, it has various pharmacological effects including antioxidant, anticancer, anti-inflammatory, anti-diabetic, and anti-atherosclerotic properties^10^. Recently, myricetin has shown neuroprotective properties by reducing neuronal damage through its antioxidant and anti-inflammatory actions^11^. In a model of middle cerebral artery occlusion, myricetin reduced ischemic brain injury by targeting mitogen-activated protein kinases (MAPK), NF-κB/p65, and AKT signaling pathways. Furthermore, myricetin improved spatial memory and depressive-like behavior in mice subjected to restraint stress, which causes cognitive decline and despair^12^. In the hippocampus, myricetin also restored the normal levels of expression of brain-derived neurotrophic factor^13^. Furthermore, it seems to directly interact with the active site of caspase-3 preventing the degeneration of primary cortical neurons. Moreover, myricetin administration enhanced the clearance of misfolded protein aggregates in neuronal cells and prevented the aggregation of various neurotoxic proteins^14^. Together, these findings support the hypothesis that myricetin has neuroprotective, antioxidant, and anti-inflammatory properties. However, it remains unclear whether myricetin can provide similar therapeutic benefits against neuroinflammation and neurological impairment following TBI. In this study, we assessed the anti-inflammatory effects of myricetin on TBI-induced CNS inflammation in rats and its potential in preventing neurological deterioration. Additionally, we assessed the changes in the epidermal growth factor receptors (EGFR)/AKT/STAT signaling pathways after myricetin treatment to elucidate the molecular mechanisms underlying its effects on TBI. Our findings reveal that myricetin increases the expression of phosphorylated (P) AKT and EGFR and inhibits the phosphorylation of STAT1/3, thus suppressing CNS inflammation and promoting the restoration of neurological function.

## Materials and methods

### Target prediction for myricetin

First, the PubChem database was searched for the 2D and 3D structures of myricetin, and SDF files containing 3D structural data were obtained. Subsequently, the SwissTargetPrediction analytical platform was used to forecast the target locations of myricetin using the obtained 3D structural files.

### Target screening for TBI

Using "Traumatic brain injury" as the keyword, the GeneCards database (https://genealacart.genecards.org/) and OMIM database (https://www.omim. org/) were searched for disease-related targets, and the results were combined to obtain a comprehensive list of TBI-related targets.

### Construction of a regulatory network of myricetin against TBI

The regulatory network of the intersection targets between myricetin and TBI was generated by mapping them one by one using Perl software. This network was subsequently visualized using Cytoscape software (V3.7.1). The network includes various forms of nodes representing the active component and target interactions.

### Protein–protein interaction network (PPI) construction and core target screening for intersecting targets

The targets of myricetin were intersected with the targets related to TBI to identify the common targets, which were considered the predicted targets of myricetin against TBI. These predicted targets were imported into the STRING online website, with the study species set as Homo sapiens and default settings applied, to generate the PPI network. Bar graphs were created based on the number of neighboring nodes connected to the targets in the PPI network.

### Kyoto encyclopedia of genes and genomes (KEGG) pathway enrichment analysis

The intersection targets obtained from the active substances and disease were processed using the Bioconductor bioinformatics package of R software^15^. This analysis enabled KEGG pathway enrichment analysis^16–18^. Statistically significant differences were set at p levels < 0.05.

### Experimental animals

The study was approved by the Experimental Animal Ethics Committee of Nantong University (approval number S20221118-019). In accordance to the ARRIVE Guidelines before and during experiments, all methods were performed in accordance with the relevant guidelines and regulations, animals were also housed in compliance with institutional guidelines of Nantong University. We purchased mature male Sprague–Dawley rats (200–250 g, approximately 2 months old) from the Animal Experiment Center of Nantong University. Sprague–Dawley rats were kept in an air-conditioned room (Temp: 22–25 °C) with standard 12 h of light/dark cycles and free access to food and water. After a period of acclimatization to the living environment, the rats used for the experiment were approximately 3 months old.

### Drug administration

Myricetin (MCE, HY-15097), lipopolysaccharide (LPS; MCE, HYD1056), EGFR inhibitor (IN) (MCE, HY144049), and AKT-IN (MCE, HY144060) were dissolved in a mixture of 10% dimethyl sulfoxide, 40% polyethylene oxide 300, 5% Tween-80, 45% saline and administered via intraperitoneal injection. Three different doses of myricetin (0, 10, and 20 mg/kg) were administered once daily from the first to the seventh day after TBI induction. LPS was added to the microglia medium at a concentration of 1 μg/ml. EGFR-IN and AKT-IN were administered according to the manufacturer's instructions.

### Development of TBI models

A controlled cortical injury (CCI) model was used to induce TBI in rat, as previously described^19^. Briefly, after successful induction of anesthesia in rats using a 2.5% mixture of isoflurane and oxygen, it was changed to a 1.5% mixture of isoflurane and oxygen to maintain anesthesia. The rat's head was then fixed in the stereotactic frame with a hot pack placed under the body to maintain body temperature at 37 °C. A median incision was made on the scalp, and a 2 mm-diameter bone window was created 2.0 mm lateral to the median sagittal line and 2.5 mm posterior to the bregma. The brain was tilted at an angle of 15° and placed perpendicular to the impactor (4-mm diameter tip; RWD Life Science, China). The impactor parameters for CCI were impact speed, 3.5 m/s; deformation depth, 1.30 mm; and duration, 400 ms. After CCI, the skin incision was sutured and treated with antibiotic ointment to prevent infection. Then, the rats were placed on a heat pad to maintain core body temperature until they had recovered from anesthesia. For the sham group, a craniotomy was also performed and the dura was exposed, but no impact was made.

### Hematoxylin and eosin (HE) staining

Rats were anesthetized with isoflurane and underwent intracardial perfusion with saline and 4% paraformaldehyde. Subsequently, their brains were extracted and dehydrated using 20% and 30% sucrose solutions. Cryosectioning was performed using a cryotome (Thermo Fisher Scientific USA). The sections were gradually rehydrated with alcohol and then rinsed with distilled water. Afterward, the sections were stained with hematoxylin for 10–15 min and counterstained with 0.5% eosin for 2–5 min. After cleaning with xylene, the sections were mounted on glass slides and dehydrated with alcohol. Glass slides and coverslips were Single Frosted Microscope Slides and EstaGlas Microscope Cover Glass purchased from CITOTEST (Nantong, China).

### Estimation of the damaged area

After Hematoxylin and eosin (HE) staining, we scanned brain slices using a fluorescent microscope (DM 5000B; Leica, Germany) and visualized the hippocampal region of the ipsilateral cortex in conjunction with rat brain atlas. Lesion areas were calculated using ImageJ (National Institutes of Health, USA) analysis software.

### Primary microglia cell culture

We used five P0-P2 neonatal rats to extract primary microglia. After aseptic processing, the head of the pup was first cut off with fine scissors. The scalp is then cut along the midline from the posterior end of the scalp to the midpoint between the eyes. When the thin skull is exposed, the end of the fine forceps is placed below the skull but above the brain tissue and pulled along the midline toward the muzzle so that the brain can be easily scooped out using curved forceps. Put the Petri dish containing the brains under a dissection microscope. Carefully remove the meninges and collect the cortices, then mince the tissue into small pieces using spring scissors^20^. After digestion with 0.25% trypsin at 37 °C for 20 min, the cells were cultured in 25 cm^2^ culture flasks covered with 0.01% PLL. The cells were grown in an F-12 medium for two weeks with regular replacement of the culture medium every two to three days. Once the cells reached confluency, the flasks were placed on a rotary shaker at 37 °C and 180 rpm for 30 min to separate microglia. The isolated microglia were then transferred to culture dishes for further studies. The purity of the isolated primary microglia confirmed using ionized calcium-binding adapter molecule 1 (IBA1) labeling, exceeded > 97%.

### Quantitative real-time PCR (qRT-PCR)

Total RNA was extracted from the cultured cells using a Total RNA kit (Vazyme, China). Reverse transcription was carried out using the Prime Script RT Reagent Kit (Takara, Japan). Reverse transcription-polymerase chain reaction (RT-PCR) was conducted using the HiScript II One-Step RT-PCR Kit (Vazyme, China). All primers for the PCR amplification were obtained from Sangon Biotech (Suzhou, China). The relative gene expression was normalized to the expression of glyceraldehyde-3-phosphate dehydrogenase (GAPDH) and analyzed using the 2^ΔΔCt method. The results were compared to those of the vehicle control. The utilized primer sequences are listed in Table 1.

**Table 1:** Sequences of primers utilized in the reverse transcription-polymerase chain reaction.

Gene	Forward primer (5–3)	Reverse primer (5–3)
iNOS	CGGACGAGACGGATAGGCAGAG	GGAAGGCAGCGGGCACATG
Cox2	AGCAGGCAGATGAAATACCAGTCT	ATACAGCTCCACAGCATCGATGT
Arg1	CTCCAAGCCAAAGTCCTTAGAG	AGGAGCTGTCATTAGGGACATC
YM1	CAGGTCTGGCAATTCTTCTGAA	GTCTTGCTCATGTGTGTAAGTGA
GAPDH	AATGGGCAGCCGTTAGGAAA	GCCCAATACGACCAAATCAGAG

Arg1, arginase 1; COX-2, cyclooxygenase-2; GAPDH, glyceraldehyde-3-phosphate dehydrogenase; iNOS, inducible nitric oxide synthase; YM1, chitinase-like protein-1.

### *Enzyme-linked immunosorbent assay* (*ELISA)*

Levels of cytokines in the rat hemisphere of the injured area and supernatant were quantified using an ELISA kit following the manufacturer's protocol (Sabbiotech, USA). The absorbance of the samples was measured at 450 nm using a microplate reader (Biotek, USA). The concentration of each sample was determined using a standard curve prepared according to the manufacturer's protocol.

### Phagocytosis assay

Immunoglobulin G antibodies labeled with fluorescein isothiocyanate-coated beads (Cayman, USA) were added to a culture dish containing microglia at a concentration of 1:1000. After an 8-h co-culture period, the cells were washed three times using a PBS solution and examined under a fluorescent microscope (DM 5000B; Leica, Germany).

### Immunofluorescence

The sections we use for immunofluorescence are frozen sections. For the antigen retrieval step, we used the Quick Antigen Retrieval Solution for Frozen Sections (Solarbio, China). Wash the sections with PBS for 5 min. Immerse the sections in Antigen Retrieval Solution and incubate at room temperature for 2 h. Wash 3–5 times for 3–5 min each with PBS to remove residual SDS and other reagents thoroughly. Subsequently, primary antibodies were incubated with the cells or brain slices overnight at 4 °C. The following day, sections were exposed to the mixture of secondary antibodies at 37 °C for an hour. To assess morphological characteristics, sections were stained with 4′,6-diamidino-2-phenylindole (Solarbio, China) for 40 min at 30 °C and examined under a fluorescence microscope (DM 5000B; Leica, Germany).

### Calcein/propidium iodide (PI) cytotoxicity assay

Microglia were seeded in 96-well plates with the appropriate amount of calcein AM/PI assay solution (1:1000) and incubated at 37 °C for 2 h. Following three washes with PBS, the staining effect was observed under a fluorescent microscope, ensuring protection from light.

### Western blot

Microglia samples were collected from culture dishes 18 h post-activation. Proteins were separated using sodium dodecyl-sulfate polyacrylamide gel electrophoresis and transferred to polyvinylidene fluoride (0.45 μm) membranes. After blocking with 5% bovine serum albumin for two hours, membranes were incubated with the primary antibody overnight. The following day, secondary antibodies (Billerica Millipore, USA) were applied to the membranes for one hour at room temperature. Protein bands were detected using an electrochemiluminescence solution. The Chemidoc detection system (Bio-Rad, USA) was used to visualize protein band signals, and ImageJ (National Institutes of Health, USA) was used to quantify them.

### Rotarod test

Motor coordination of rats was evaluated using the accelerated Rotarod test before and on days 1, 7, and 14 after TBI. The test involved a rotating rod with a 10 cm diameter. Within 60 s, the spindle speed was increased from 4 to 30 rpm and remained constant at 30 rpm for 300 s. A sensor was employed to detect the moment when the rat stumbled and fell off the spindle.

### Morris water maze

Morris water maze is a behavioral test used to evaluate neurological function in rats. The experiment consisted of two phases: training and testing. The platform was positioned and installed in the middle of the third quadrant of a circular pool. Rats were gently released into the pool from each of the four quadrants facing the pool's wall. The distance and time taken (escape latency) from entering the water to arriving at the platform were measured. On the fourth day, the test phase was conducted. The platform was removed from the water tank, and each rat was put in the pool at the location where the platform was originally positioned. The swimming trajectory of the rats was tracked for two minutes and recorded on a computer.

### Modified neurological severity score (mNSS)

To assess the neurological function in rats, the mNSS was assessed on days 1, 7, and 14 after TBI. The mNSS tests comprise a combination of motor, sensory, balance, and reflex assessments. A score of one point is assigned for each absent reflex or abnormal response. Thus, on a scale of 0–18, a score of 0 indicates normal neurological function without deficits, while a score of 18 indicates severe neurological deficits^21^.

### Randomization and blinding

All animals were randomly assigned to groups for inclusion in the analysis. The operators performing the experiments and analyzing the data analysis who performed the experiments and data analysis were not aware of the distribution throughout the experiment.

### Antibodies

For immunofluorescence staining, the following primary antibodies were employed: rabbit /mouse anti-glial fibrillary acidic protein (GFAP) (1:500 ab7260 ab279290, Abcam), rabbit/mouse anti-IBA1 (1:500 ab178846 ab283319, Abcam), rabbit anti-CD86 (1:500 MA5-32078, Thermo-Fisher), and rabbit anti-CD206 (1:500 PA5-101657, Thermo-Fisher). The secondary antibodies comprised: Alexa Fluor® 488 donkey anti-mouse IgG (1:1000, ab150113, Abcam), Alexa Fluor® 488 donkey anti-rabbit IgG (1:1000 ab150077, Abcam), Alexa Fluor® 594 donkey anti-mouse IgG (1:1000, ab150116, Abcam), and Alexa Fluor® 594 donkey anti-rabbit IgG (1:1000, ab150080, Abcam).

For Western blot, the following antibodies were employed: rabbit anti-P-STAT1 (1:1000, ab109461, Abcam), rabbit anti-STAT1 (1:1000, ab234400, Abcam), rabbit anti-P-STAT3 (1:500, PA5-17,876, Thermo-Fisher), mouse anti-STAT3 (1:500, ab68153, Abcam), and mouse anti-P-AKT (1:500, ab283852, Abcam), rabbit anti-AKT (1:500, ab283852, Abcam), rabbit anti-EGFR (1:500, ab525894, Abcam), and rabbit anti-GAPDH (1:2000, ab9485, Abcam).

### Statistical analysis

GraphPad Prism software v. 9.1.4 was used to analyze the data, which were presented as mean ± standard deviation. Unpaired Student's t-test, one-way ANOVA, and two-way ANOVA were used to compare the data. P-values ≤ 0.05 indicated statistically significant differences. All experiments were repeated at least thrice.

### Ethical approval

Animal experiments were approved by the Experimental Animal Ethics Committee of Nantong University. (S20221118-019).

## Results

### Molecular structure of myricetin and general design of the experiment

**Figure 1:**
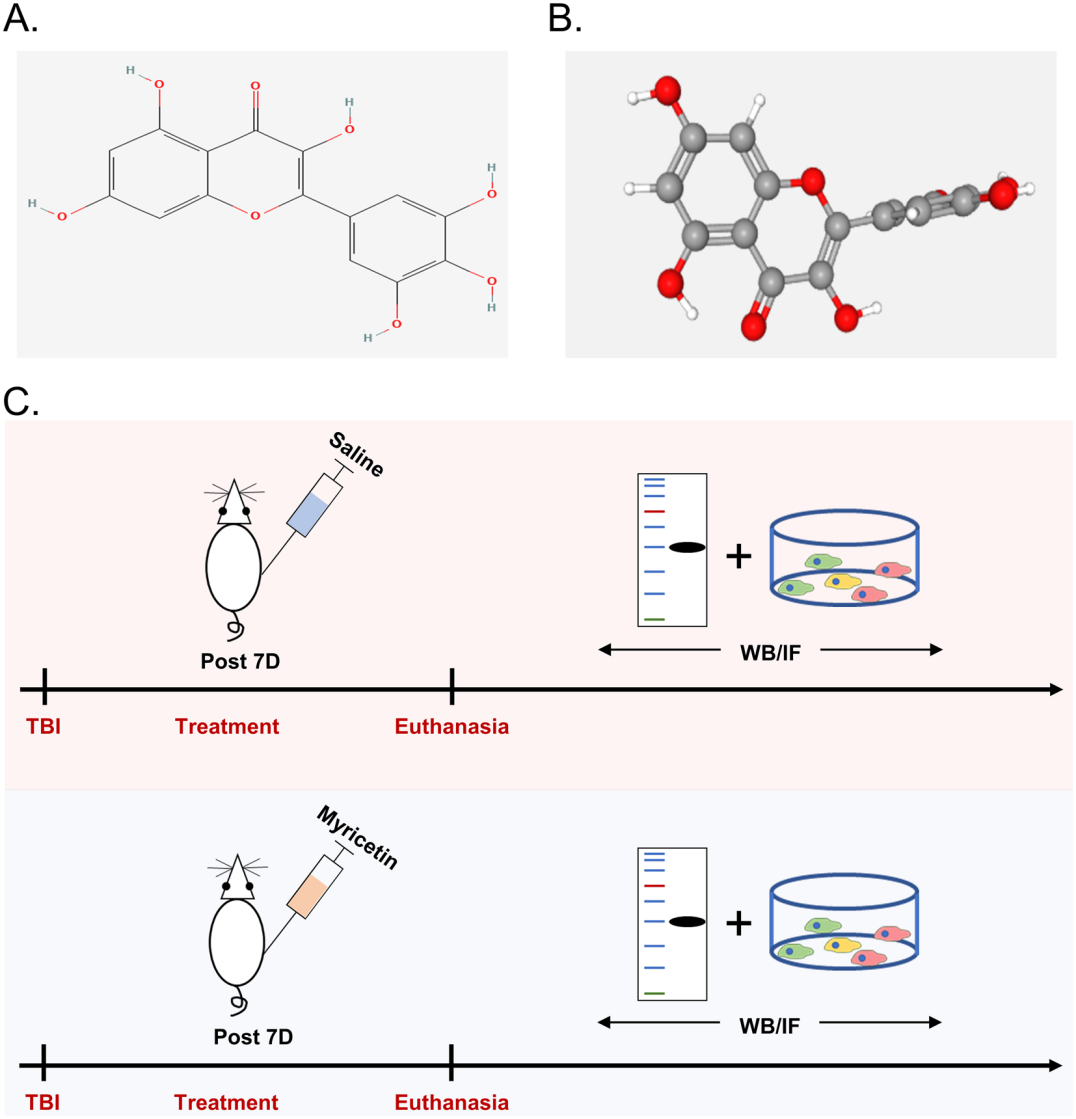
Structure of myricetin and experimental design of the study. (**A**) 2D structure of myricetin. (**B**) 3D structure of myricetin. (**C**) General experimental design.

Myricetin is a naturally occurring flavonoid. Myricetin's two- and three-dimensional chemical structures are shown in Fig. 1A,B. According to studies using several models of neurodegenerative disorders like Alzheimer's and Parkinson's, myricetin exhibits good preventive properties^14^. For example, myricetin inhibits Aβ1-40, Aβ1-42 and α-synuclein aggregation, including their oligomerizations, which may have a protective effect against AD and Parkinson's disease. In addition, myricetin may also exert anti-PD effects by increasing tyrosine hydroxylase expression in the striatum and inhibiting pathology associated with iron accumulation in the substantia nigra. Whether this compound has the same therapeutic efficacy against excessive neuroinflammation and neurological decline after TBI is still unknown, so we designed a series of in vivo experiments to examine the therapeutic effect and mechanism of myricetin using a rat model of TBI (Fig. 1C).

### Myricetin promoted the healing of the TBI area, inhibited inflammatory cell infiltration in the injury area, and promoted the recovery of nerve function

**Figure 2:**
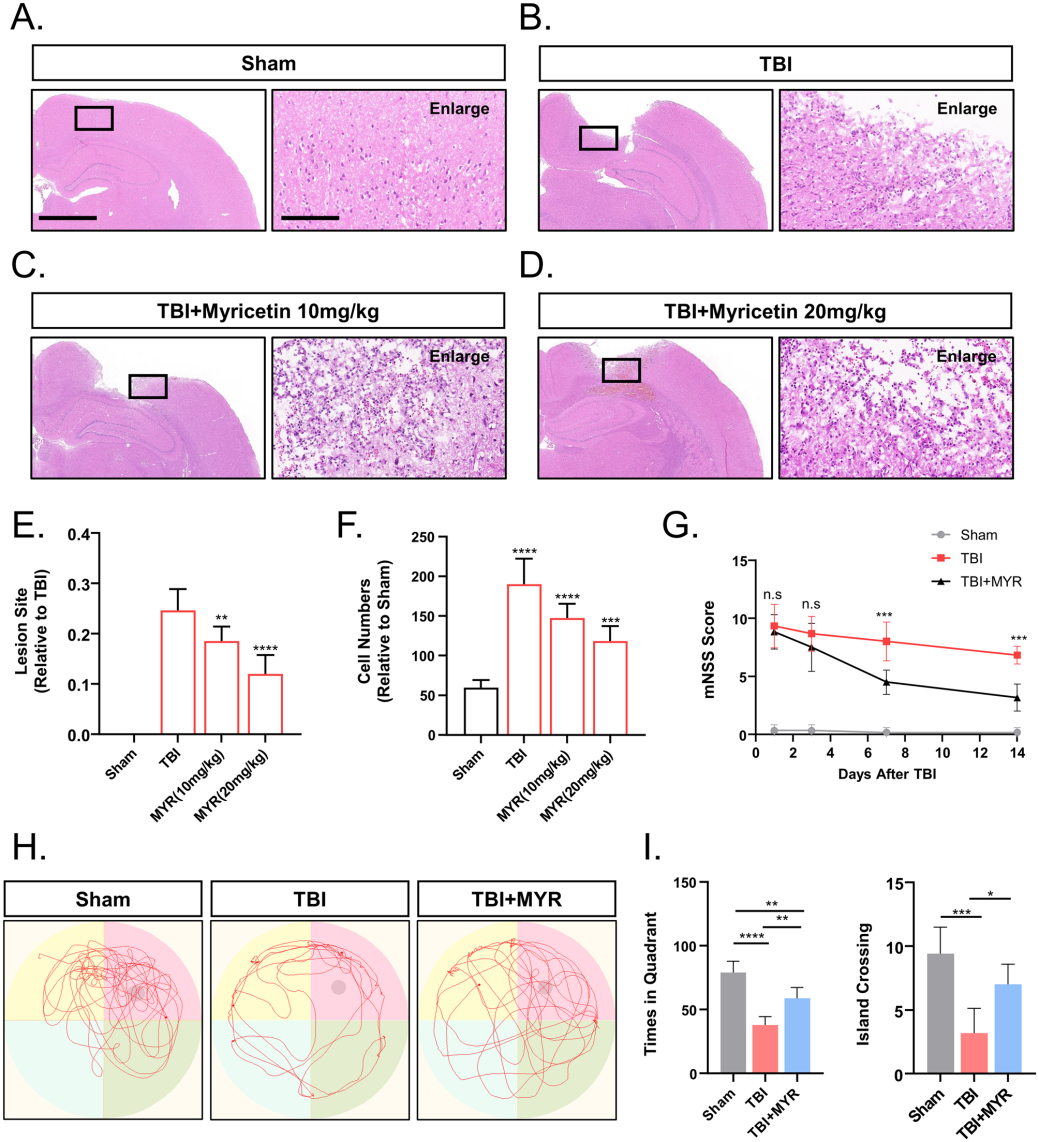
Myricetin inhibits neuroinflammation and promotes neurological recovery in rats with TBI. (**A**–**D**) Hematoxylin and eosin staining showing that myricetin promoted wound healing and inhibited the infiltration of inflammatory cells. (**E**) Quantification of damage area expressed as a proportion of the cerebral hemisphere. (**F**) Quantitative analysis of the differences in the number of infiltrating inflammatory cells in the injury area. (**G**) Modified neurological severity score in the saline- and myricetin- treated groups. (**H**) Representative results of the Morris water maze in the saline- vs. myricetin-treated groups. (**I**) Quantitative statistics of the differences in the time spent in the target quadrant and number of island crossings. Scale bar = 2.5 mm and 100 um. n.s., p > 0.05, **p ≤ 0.01, ***p ≤ 0.001, ****p ≤ 0.0001. The results are presented as mean ± standard deviation (n = 5/6).

With the results of the pre-experiment, we found that microglia and astrocytes responded most vigorously on the seventh day after traumatic brain injury (Supplementary Fig. 1). Previous studies have shown that myricetin at doses of 10 and 20 mg/kg has a better therapeutic effect on rats with cerebral ischemia^12^. Therefore, we divided the rats into 4 groups: sham-group, TBI group, 10 mg/kg myricetin-treated group, and 20 mg/kg myricetin-treated group to investigate the ideal dose of myricetin for the treatment of TBI in rats (Fig. 2A–D). Compared to control, myricetin improved wound healing (Fig. 2E), and reduced the number of infiltrating inflammatory cells in the injury area (Fig. 2F) in a dose-dependent manner. As the 20 mg/kg dose had the better therapeutic effect, we selected that concentration for the follow-up study.

mNSS scores are commonly used as a reliable indicator of neurological function in rats. Rats in the myricetin-treated group exhibited a rapid and significant decrease in mNSS scores compared to untreated rats (Fig. 2G).

The results of the Morris water maze showed that myricetin exhibited a favorable therapeutic effect on TBI (Fig. 2H). The myricetin-treated group exhibited better neurological recovery compared to the saline-treated group, as demonstrated by longer dwell time in the target quadrant and increased island crossings (Fig. 2I). These findings supported the efficacy of myricetin treatment in the rat model of TBI.

Intraperitoneal injection of myricetin limited the infiltration of inflammatory cells in the injury area and promoted the recovery of learning and memory functions in rats with TBI. Thus, the brain damage area recovered significantly faster in myricetin-treated rats than in saline-treated rats.

### Myricetin inhibits the activation of microglia and astrocytes in brain injury areas

**Figure 3:**
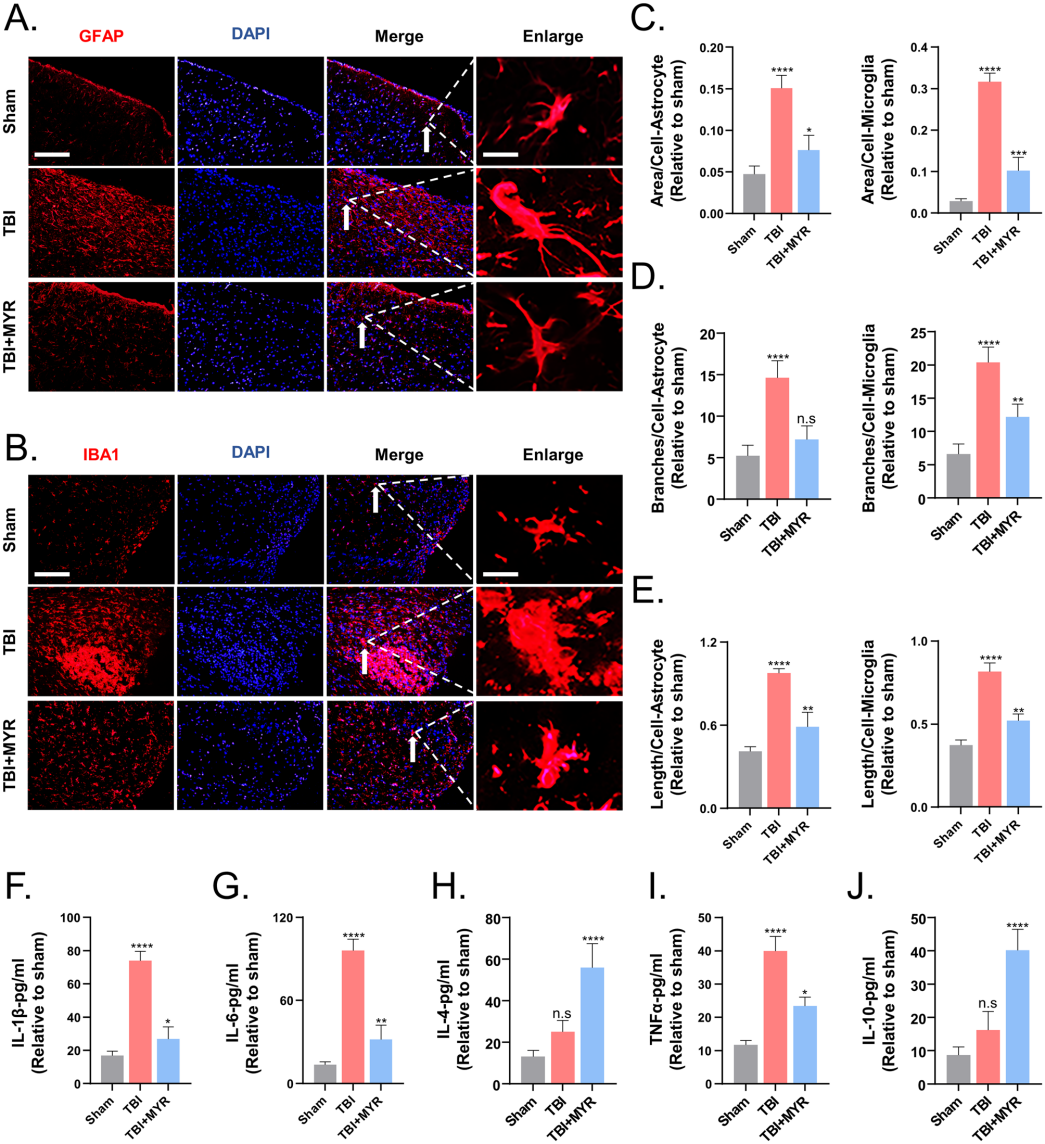
Myricetin inhibits the inflammatory response of microglia as well as astrocytes after TBI. Immunofluorescence results representative for the myricetin-induced inhibition of the inflammatory response of (**A**) astrocytes and (**B**) microglia, after TBI. (**C**) Statistics of the number of infiltrating astrocytes and microglia in the lesion area. (**D**) Number of microglia and astrocyte branches in the lesion area. (**E**) Quantification of the longest diameter of microglia and astrocytes in the lesion area. (**F**) Levels of expression of IL-1β, IL-6, IL-4, TNF-α, and IL-10 in the lesion area—ELISA. Scale bar = 200 um and 10 um. n.s., p > 0.05, *p ≤ 0.05, **p ≤ 0.01, ***p ≤ 0.001, ****p ≤ 0.0001. The results are presented as mean ± standard deviation (n = 5).

Astrocytes and microglia, the two most abundant glial cells in the CNS, play crucial roles in shaping the inflammatory microenvironment in brain injury areas. To this end, we used immunofluorescence to assess the expression of GFAP, an astrocyte marker, and IBA1, a microglia marker. Following TBI, the number of microglia and astrocytes in the injury area increased rapidly (Fig. 3A,B). Furthermore, changes in their morphology were observed.

Using ImageJ software, we quantified the injured area (Fig. 3C), the number of branches (indicating structural complexity) (Fig. 3D), and the diameter (Fig. 3E) of microglia and astrocytes in the injury area. The analysis revealed a rapid increase in both microglia and astrocytes, along with a more complex topology, after TBI compared to sham-procedure.

After myricetin treatment, microglia and astrocytes in the injury area exhibited morphology similar to the one observed in the resting state, which was supported by statistical data. Additionally, we dissected the brain of rats with TBI and performed ELISA after homogenization to determine the peri-wound levels of cytokines. The results indicated that TBI determined a significant increase in the levels of classical pro-inflammatory cytokines such as IL-1β, IL-6, and TNF-α. Furthermore, the levels of anti-inflammatory cytokines such as IL-4 and IL-10 did not increase significantly after the injury, indicating an extreme local enhancement of inflammation. Administration of myricetin suppressed the TBI-induced excessive inflammatory response. Compared to the control, myricetin decreased the levels of expression of IL-1β, IL-6, and TNF-α while increasing the levels of expression of the anti-inflammatory cytokines IL-4 and IL-10. These results indicated a reduction of the inflammatory state in the injured area, contributing to the quiescence of inflammation and recovery of neurological function (Fig. 3F).

### Myricetin enhances the neuroprotective functions of microglia

**Figure 4:**
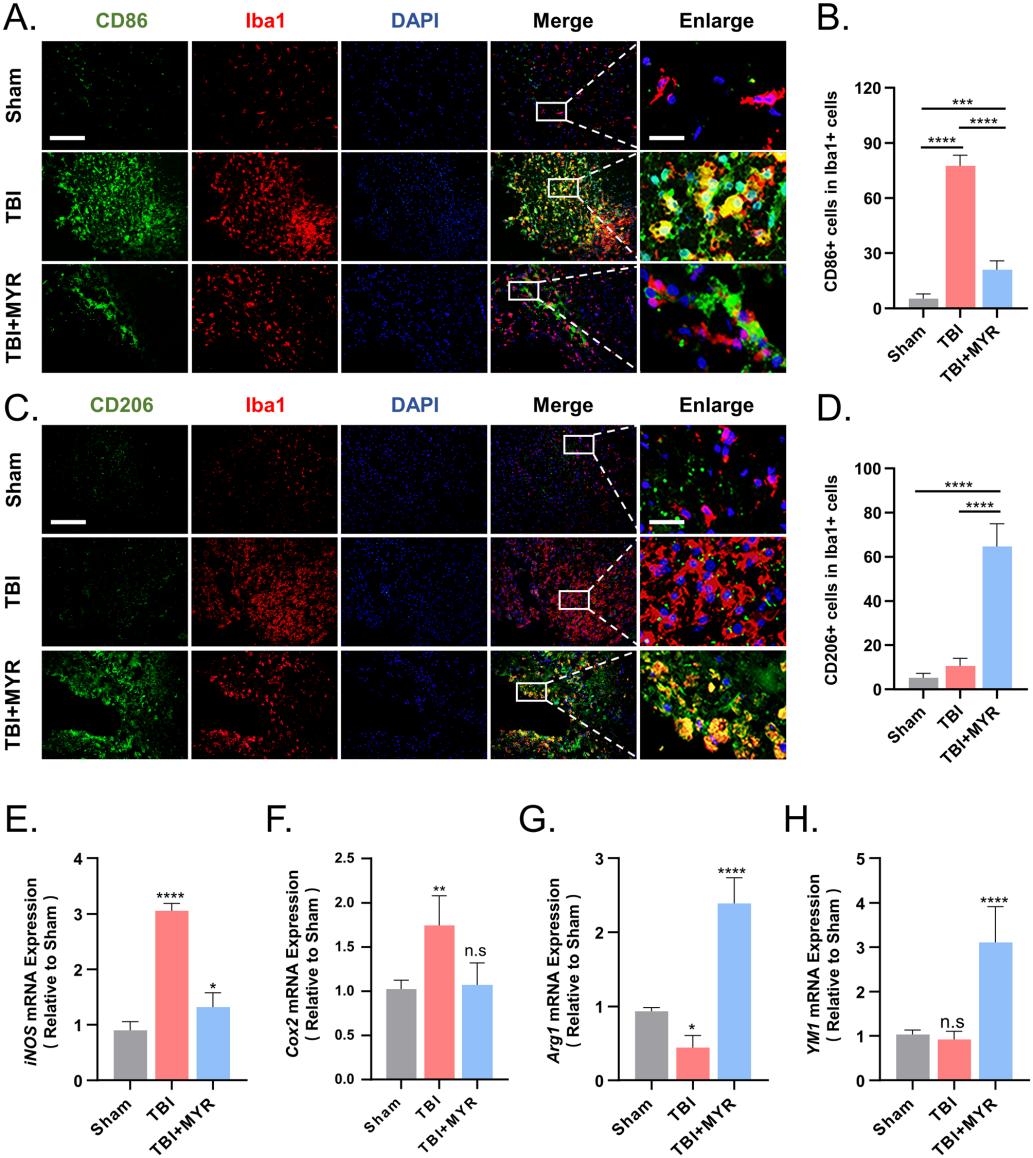
Myricetin impacts the polarization state of microglia in the lesion area. (**A**) Immunofluorescence results representative for the myricetin-induced inhibition of the microglial expression of CD86 expression in the lesion area. (**B**) Quantitative statistics of the differences in the %area of thresholded IBA1 occupied by thresholded CD86. (**C**) Immunofluorescence results representative for myricetin-induced enhancement in the microglial expression of CD206 in the lesion area. (**D**) Quantitative statistics of the differences in the %area of thresholded IBA1 occupied by thresholded CD206. (**E**–**H**) Quantitative statistics of the differences in the levels of expression of iNOS, COX-2, Arg1, and YM1 detected by quantitative real-time polymerase chain reaction. Scale bar = 200 um and 50 um. n.s., p > 0.05, *p ≤ 0.05, **p ≤ 0.01, ***p ≤ 0.001, ****p ≤ 0.0001. The results are presented as mean ± standard deviation (n = 5).

CD86, a marker of toxic microglia, indicates that microglia are promoting neuroinflammation and cell killing. In the injury area, the presence of CD86 and IBA1 double-positive cells indicates a highly pro-inflammatory state following TBI. However, the administration of myricetin significantly improved the inflammatory response in the injury area, allowing over-activated microglia to rest (Fig. 4A,B).

We also investigated the expression of CD206, a marker associated with neuroprotective microglia, in the injured brain tissue. Immunofluorescence results indicated no significant difference in the expression of CD206 after traumatic brain injury, suggesting an unbalanced inflammatory state in the injured area. Compared to saline treatment, myricetin treatment decreased the number of microglia while increasing the expression of CD206 (Fig. 4C,D). The shift towards a neuroprotective phenotype in microglia facilitated the suppression of inflammation and neural repair in the injury zone.

To strengthen our findings, we performed qRT-PCR on brain tissue from the injury area, analyzing four microglia functional markers: iNOS, COX-2, arginase 1 (Arg1), and chitinase-like protein-1 (YM1). These results were also consistent with our hypothesis^22^. Treatment with myricetin reduced the expression of neurotoxic microglia markers, iNOS and COX-2, while increasing the expression of neuroprotective microglia markers, Arg1 and YM1 (Fig. 4E–H). These results indicate that myricetin significantly reduces inflammation induced by microglia infiltrated in the injury area and promotes the repair of neurological functions in rats with TBI.

### *Myricetin inhibits microglial inflammation and apoptosis *in vitro

To assess the polarization status and apoptosis of microglia, we assessed the levels of P-STAT1/3 and P-AKT respectively, using immunofluorescence and live-dead cell assay^23–25^.

In vitro, we induced intense inflammation in microglia by stimulating them with LPS, a component of the outer wall of Gram-negative bacterial cells that activates microglia via TLR4. LPS was administered at a dosage of 100 ug/mL, and subsequent experiments were performed after 8 h of stimulation.

**Figure 5:**
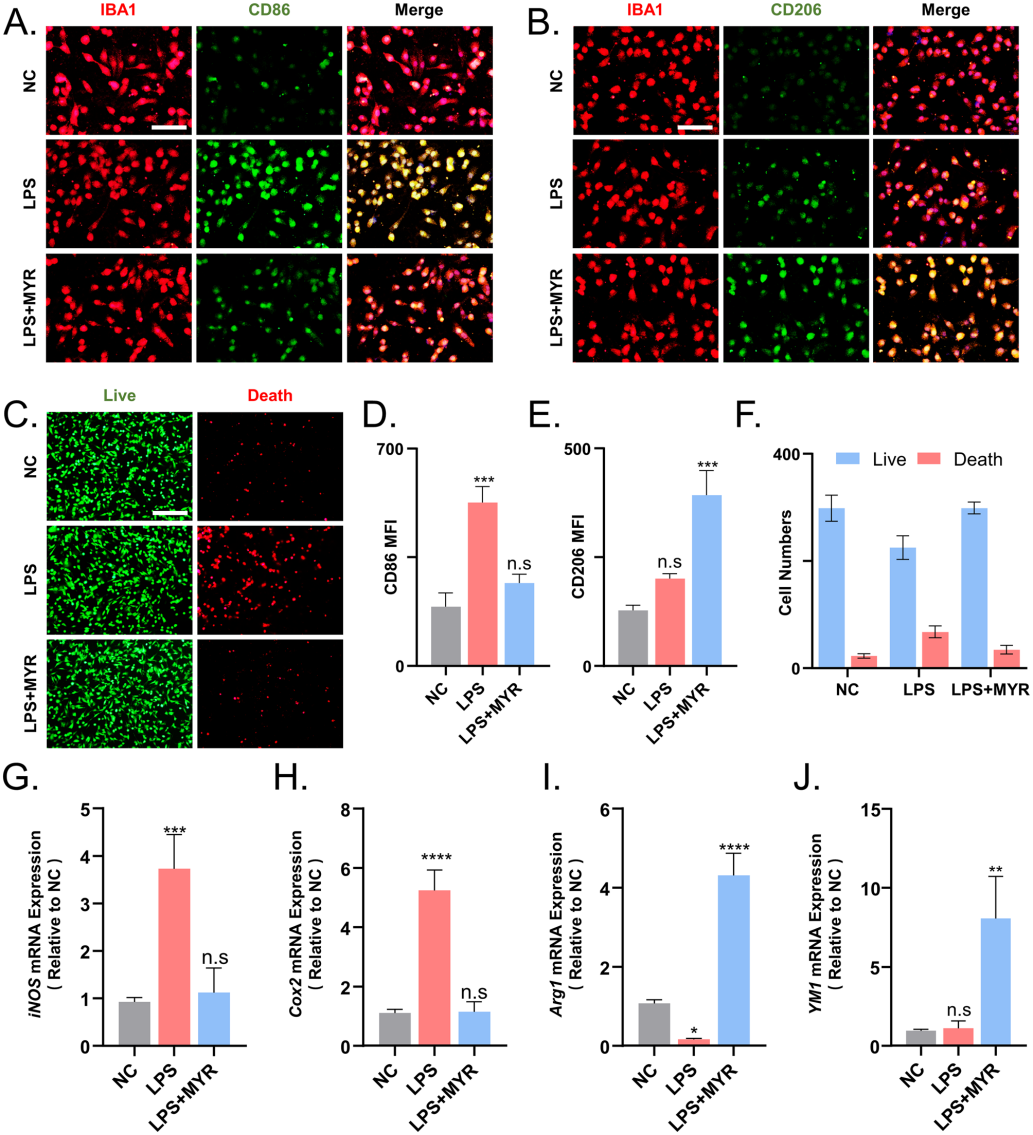
Myricetin inhibits lipopolysaccharide (LPS)-induced microglial inflammation and apoptosis in vitro. Immunofluorescence results showing the inhibitory effect of myricetin on LPS-induced (**A**) microglial expression of CD86, (**B**) decrease in the microglial expression of CD206, and (**C**) apoptosis in microglia. (**D**) Quantitative statistics of the differences in the mean fluorescence intensity of CD86. (**E**) Quantitative statistics of the differences in the mean fluorescence intensity of CD206. (**F**) Quantitative statistics of the differences in the number of live and death cells. (**G**–**J**) Quantitative analysis of the differences in the expression levels of *iNOS*, *Cox2*, *Arg1,* and *YM1* in microglia determined using quantitative real-time polymerase chain reaction. Scale bar = 200 um and 50 um. n.s., p > 0.05, *p ≤ 0.05, **p ≤ 0.01, ***p ≤ 0.001, ****p ≤ 0.0001. The results are presented as mean ± standard deviation (n = 3).

Immunofluorescence results indicated that myricetin significantly reduced apoptotic cells and increased the level of expression of CD206 in microglia (Fig. 5A,B, and D,E). Furthermore, the cells exposed to myricetin maintained their viability (Fig. 5C,F).

We also performed qRT-PCR to assess the expression of *iNOS*, *Cox2*, *Arg1*, and *YM1* in microglia cultured in vitro. The results indicated that myricetin effectively suppressed the inflammatory state of microglia (Fig. 5G–J).

### Myricetin targets EGFR and AKT to promote inflammatory quiescence

**Figure 6:**
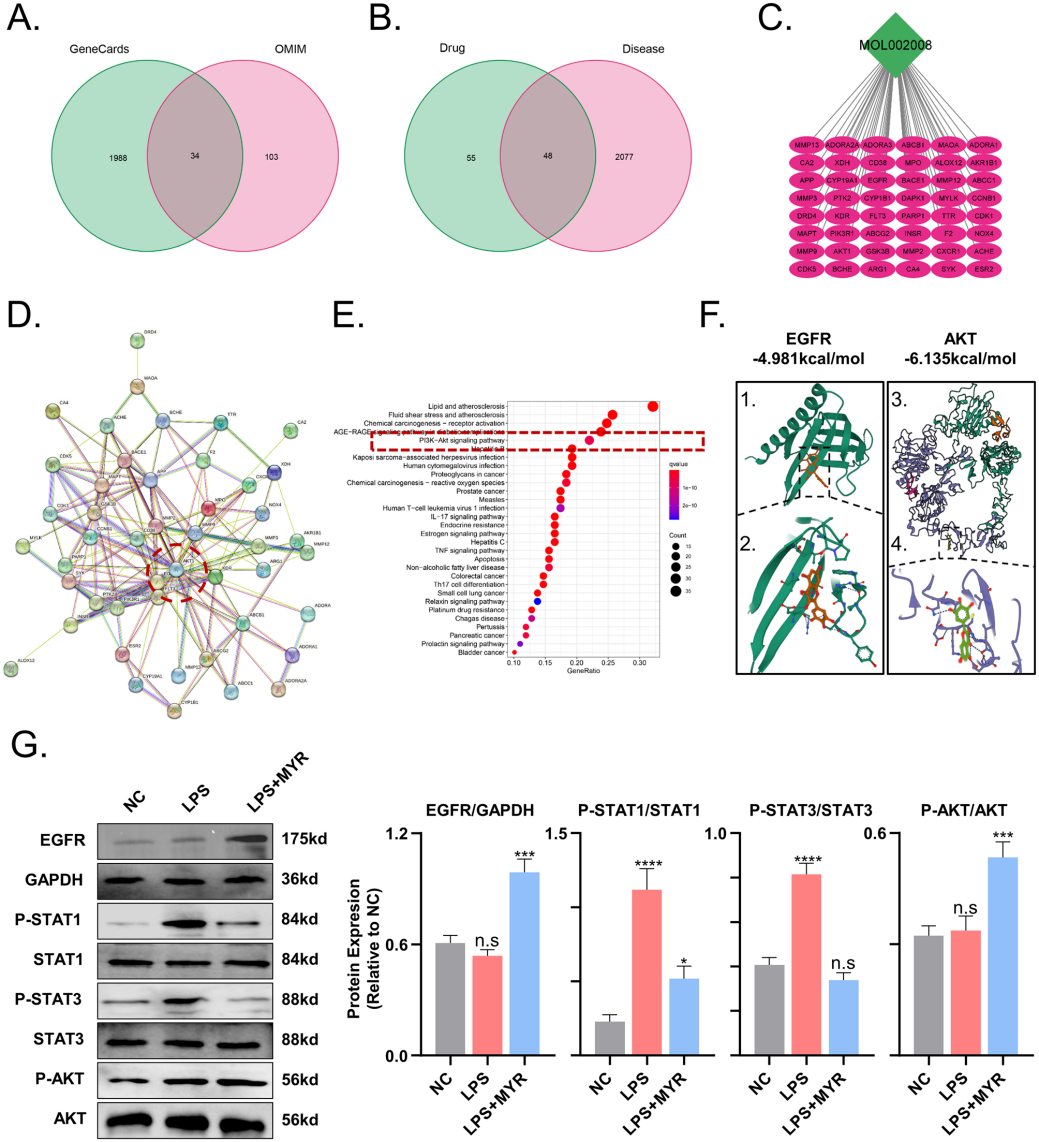
Myricetin targets the EGFR-AKT/STAT pathway to inhibit neuroinflammation. (**A**) Venn diagram of TBI targets. (**B**) Venn diagram of drug-disease intersection targets. (**C**) Regulatory network of myricetin's interaction with targets. (**D**) Protein interaction network of key targets. (**E**) Barplot depicting KEGG pathway enrichment analysis. (**F**) Molecular docking of myricetin with EGFR and AKT. (**G**) Representative Western blot results for EGFR, P-STAT1, P-STAT3, P-AKT, and corresponding quantitative analysis. n.s., p > 0.05, *p ≤ 0.05, **p ≤ 0.01, ***p ≤ 0.001, ****p ≤ 0.0001. The results are presented as mean ± standard deviation (n = 3).

Using "Traumatic brain injury" as a keyword, we searched the GeneCards and OMIM databases and obtained 2125 targets. After intersecting these targets with 103 targets of myricetin using Perl software (Fig. 6A–C), we obtained a list of 48 intersecting targets. Analysis of the PPI network revealed that AKT1 and EGFR are located at the core of the network with the highest number of neighboring nodes, suggesting their importance in myricetin’s role in TBI prevention and treatment (Fig. 6D). KEGG pathway enrichment analysis identified seven pathways with substantial enrichment, including IL-17 signaling, tumor necrosis factor signaling, estrogen signaling, Th17 cell differentiation, apoptosis, and PI3K-Akt signaling pathways (Fig. 6E).

Molecular docking studies using Autodock Vina v.1.2.2^26^, demonstrated the affinity of myricetin for EGFR and AKT. The binding energy analysis revealed stable interactions, with low binding energies of −4.981 and −6.135 kcal/mol for EGFR and AKT, respectively (Fig. 6F).

To validate these results, we performed Western-Blot assay. Protein samples were collected from the saline-treated (control), LPS-treated, and LPS and myricetin co-treated groups. Western blot analysis indicated that myricetin significantly increased the expression level of EGFR and phosphorylation of AKT, and effectively inhibited the phosphorylation of STAT1 and STAT3, two transcription factors associated with toxic glial cells. These results, along with the molecular docking findings, demonstrate that myricetin exerts an anti-inflammatory and neuroprotective role in the TBI rat model through the EGFR-AKT/STAT pathway (Fig. 6G).

### *Inhibition of EGFR and AKT expression impairs the therapeutic effect of myricetin on microglia *in vitro

**Figure 7:**
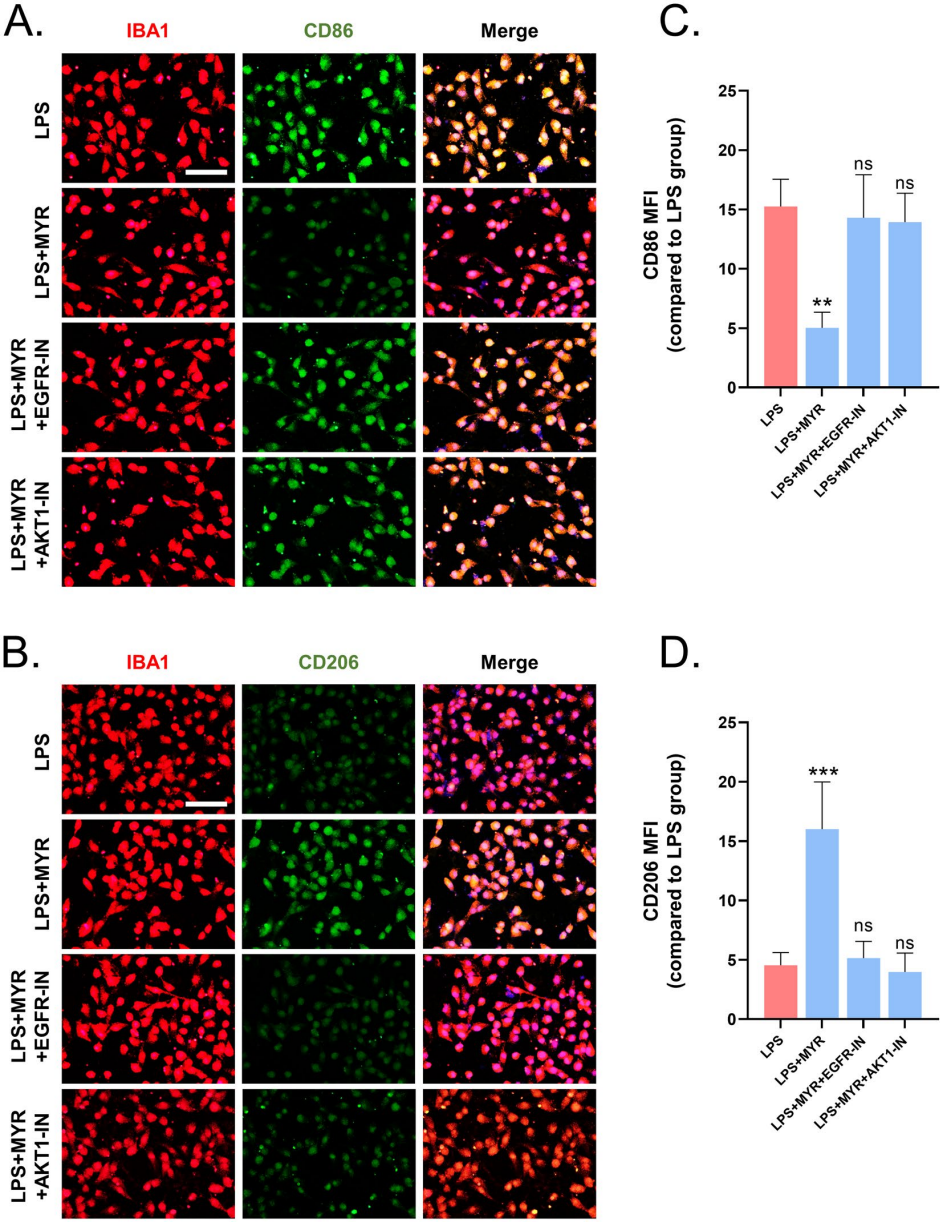
Inhibitors of EGFR (EGFR-IN) and AKT (AKT-IN) prevent the therapeutic effect of myricetin on lipopolysaccharide (LPS)-stimulated microglia. Representative immunofluorescence results of (**A**) CD86 expression, and (**B**) CD206 expression, following treatment with EGFR-IN or AKT-IN and myricetin. Quantitative statistics of the differences in the mean fluorescence intensity of (**C**) CD86, and (**D**) CD206. Scale bar = 200 um and 50 um. n.s., p > 0.05, *p ≤ 0.05, **p ≤ 0.01, ***p ≤ 0.001, ****p ≤ 0.0001. The results are presented as mean ± standard deviation (n = 3).

To further clarify the molecular mechanism underlying the neuroprotective effect of myricetin, we exposed LPS-stimulated microglia treated with myricetin to EGFR-IN and AKT-IN. The results revealed that both EGFR-IN and AKT-IN effectively disrupted the therapeutic effect of myricetin, as evidenced by increased levels of expression of CD86 and decreased levels of expression of CD206 (Fig. 7A–D).

**Figure 8:**
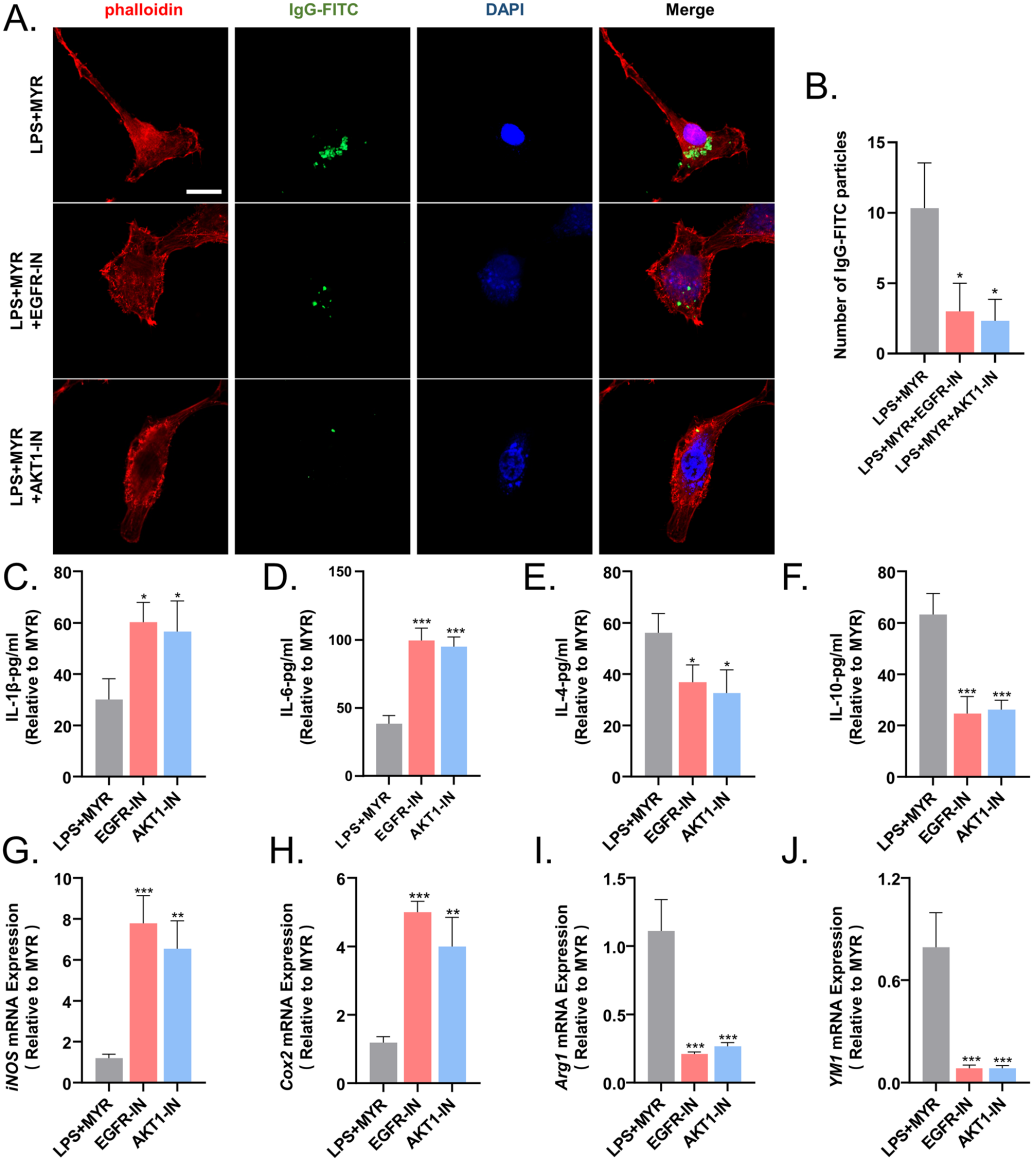
Inhibitors of EGFR (EGFR-IN) and AKT (AKT-IN) prevent the inhibition of microglial inflammation by myricetin. (**A**) Inhibition of microglial phagocytosis by EGFR-IN and AKT-IN. (**B**) Quantitative statistics of the differences in the mean fluorescence intensity of immunoglobulin G antibodies labeled with fluorescein isothiocyanate. (**C**–**F**) Microglial levels of expression of IL-1β, IL-6, IL-4 and IL-10 assessed using ELISA. (**G**–**J**) Quantitative analysis of the expression levels of *iNOS*, *Cox2*, *Arg1,* and *YM1* in microglia determined using qRT-PCR. n.s., p > 0.05, *p ≤ 0.05, **p ≤ 0.01, ***p ≤ 0.001, ****p ≤ 0.0001. The results are presented as mean ± standard deviation (n = 3).

Phagocytosis by microglia is correlated with the inhibition of inflammation and neurological functional recovery. In vitro IgG particle phagocytosis assays showed that inhibition of EGFR and AKT significantly impaired the phagocytosis of microglia (Fig. 8A,B). Additionally, the levels of expression of inflammatory cytokines (Fig. 8C–F) and microglial cytotoxic makers re-escalated upon EGFR and AKT inhibition (Fig. 8G–J).

### Inhibition of EGFR and AKT expression disrupts the neuroprotective effect of myricetin in rats with TBI

**Figure 9:**
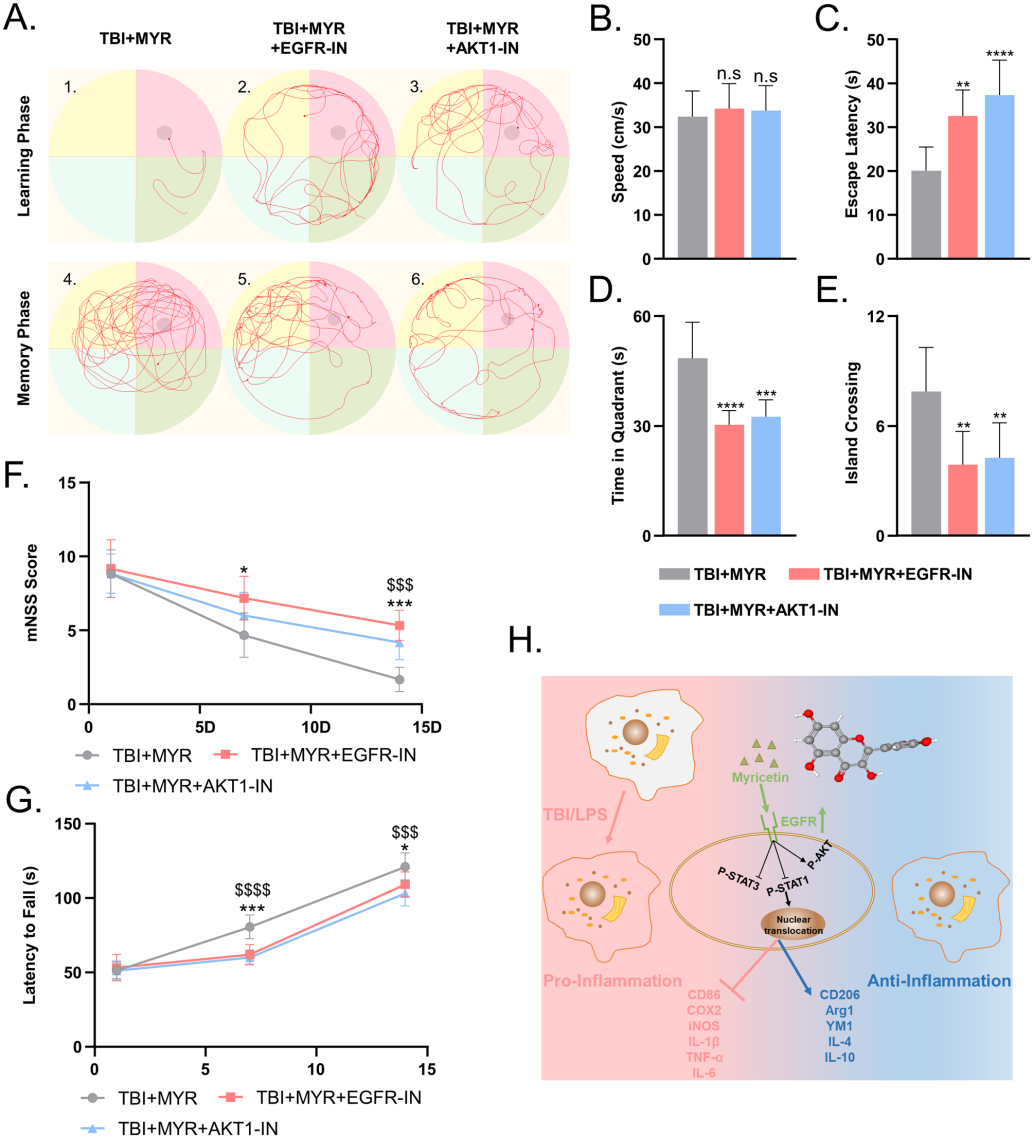
Inhibitors of EGFR (EGFR-IN) and AKT (AKT-IN) prevent the neurofunctional repair induced by myricetin in rats with TBI. Representative results of (**A**–**E**). Morris water maze, (**F**). Modified neurological severity score (mNSS), and (**G**). Rotarod test, indicating the inhibition of neural function repair by EGFR-IN and AKT-IN in rats with TBI. (**H**). Graphical abstract summarizing the overall results of this study. n.s., p > 0.05, *p ≤ 0.05, **p ≤ 0.01, ***p ≤ 0.001, ****p ≤ 0.0001. The results are presented as mean ± standard deviation (n = 6).

We used the mNSS score, as well as Morris water maze and Rotarod tests to fully evaluate the neurological function of rats with TBI treated with myricetin and EGFR-IN or AKT-IN. The results of the Morris water maze test (Fig. 9A–E), the mNSS score (Fig. 9F), and the results of the Rotarod test (Fig. 9G) indicated that EGFR-IN and AKT-IN disrupted the beneficial effects of myricetin on neurological recovery in rats.

## Discussion

TBI is one of the most commonly encountered traumatic diseases in neurosurgery, often caused by traffic accidents, falls, and heavy objects injuries due to urbanization and increased transportation. The treatment of TBI is costly, and the overall prognosis is poor, placing a heavy burden on society and affected families^1^. Furthermore, no drugs are available to significantly improve the prognosis of TBI. The clinical trials investigating the anti-TBI efficacy of experimental drugs, such as minocycline^27^ and glucocorticoids^28^, have not shown satisfactory results. Therefore, the development of novel drugs for TBI is a current research priority in neuropharmacology^29^. Although the pathophysiological mechanisms of TBI are not fully understood, emerging evidence highlights the important role of excessive neuroinflammation in the pathophysiology following TBI.

The crucial role of microglia in the immune response of the CNS is widely acknowledged. Nevertheless, excessive microglial activation following TBI leads to the release of pro-inflammatory cytokines and neurotoxic substances, contributing to treatment failure and adverse mental effects^4^. Therefore, targeting microglial responses to suppress excessive neuroinflammation may promote the recovery of patients with TBI. COX-2 and iNOS are stimulated by several CNS diseases. iNOS-derived nitric oxide (NO) is a key regulator of neuroinflammation, promoting vasodilation and facilitating inflammatory cell infiltration at the site of injury. Moreover, high levels of NO also induce the expression of COX-2, which catalyzes the conversion of arachidonic acid into PGE2, a neuroinflammatory mediator implicated in the emergence of TBI complications^30,31^. Thus, the development of therapeutic interventions for TBI has focused on targeting and inhibiting the synthesis and activity of these regulators.

As a natural flavonoid, myricetin was initially of interest for its antioxidant and free radical scavenging activity. Recently, in an in vitro experiment with an oxygen–glucose deprivation (OGD) model in HSY5Y cells, it was demonstrated that direct binding and inhibition of myricetin to caspase-3 reduced reactive oxygen species production, mitochondrial depolarization and neuronal damage^12^. And in the MCAO rat model, myricetin was shown to reduce neuronal apoptosis and infarct size by inhibiting abnormally increased pro-inflammatory cytokines and reactive oxygen species^32^. In addition, myricetin reversed streptozotocin-induced reduction of hippocampal neurons or D-galactose-induced reduction of ERK1/2 and CREB phosphorylation levels to ameliorate learning and memory deficits in a rodent model of AD^33,34^. In summary, many studies have shown that myricetin has favorable pharmacological effects on neurological disorders.

Our results demonstrate that myricetin therapy effectively reduces the TBI-induced expression of pro-inflammatory cytokines, including IL-1, IL-6, and TNF-α, as well as that of iNOS and COX-2 in the injured brain of rats. Similar therapeutic effects were observed in vitro using LPS-stimulated microglia. Myricetin treatment significantly upregulated the expression of neurorestorative microglia markers such as CD206, Arg1, and YM1, while increasing the expression of anti-inflammatory cytokines, such as IL-4 and IL-10, in the injured area. These findings suggest that myricetin treatment effectively counteracts the inflammatory state in the brain injury area, promotes the functional transformation of microglia, regulates the excessive inflammatory response, and enhances the recovery of neurological functions in rats with TBI.

In this study, we demonstrated that myricetin inhibits pro-inflammatory mediators by interfering with STAT1/3 phosphorylation. Furthermore, it promoted AKT phosphorylation and EGFR expression, inhibiting apoptosis, and enhanced the production of IL-4 and IL-10, two anti-inflammatory cytokines which prevent excessive neuroinflammation following TBI.

These findings suggest that myricetin has potential as a neuroprotective drug by reducing inflammation and apoptosis in TBI. Furthermore, due to its high lipophilia, myricetin crosses the blood–brain barrier, which further supports its potential use in treating TBI-induced persistent brain dysfunction^35^. However, our study has some limitations. We did not examine the effects of myricetin on centrally infiltrated adaptive immune cells such as T and B cells, which play a role in the chronic course of TBI^36^. Therefore, further research is needed to confirm the long-term therapeutic effects of myricetin in patients with TBI.

## Conclusion

In conclusion, the present study demonstrated that myricetin has anti-inflammatory and neuroprotective effects after brain injury, potentially through regulating the phosphorylation levels of AKT and STAT1 and the EGFR-AKT/STAT signaling pathway. Furthermore, it promotes neurological recovery after TBI. Thus, myricetin may be a therapeutic agent with the potential for clinical translational.

### Supplementary Information

Supplementary Information 1.

Supplementary Information 2.

Supplementary Information 3.

Supplementary Information 4.

## Data Availability

Raw data requests from anyone should be directed to the corresponding authors, ntgpp@ntu.edu.cn or 2013310106@stmail.ntu.edu.cn. Data will be available on reasonable request.
