# Supplementary material for: Myricetin suppresses traumatic brain injury-induced inflammatory response via EGFR/AKT/STAT pathway
Source: Sci Rep. 2023 Dec 20;13:22764. doi: 10.1038/s41598-023-50144-x (PMC10733425; doi:10.1038/s41598-023-50144-x)
Supplement: Supplementary file 1 — Supplementary Information 1. [file 41598_2023_50144_MOESM1_ESM.docx]

**Scientific Reports**

**Supplementary Information**

**Myricetin suppresses traumatic brain injury-induced inflammatory response via EGFR/AKT/STAT pathway**

1 Department of Neurosurgery, Affiliated Hospital of Nantong University, Medical School of Nantong University, Nantong, Jiangsu, 226001, China.

2 Research Center of Clinical Medicine, Affiliated Hospital of Nantong University, Nantong, Jiangsu, 226001, China.

&These authors contribute equally to this work

*Correspondence Authors

Peipei Gong: ntnsgpp@163.com

Zhichao Lu: 2013310106@stmail.ntu.edu.cn


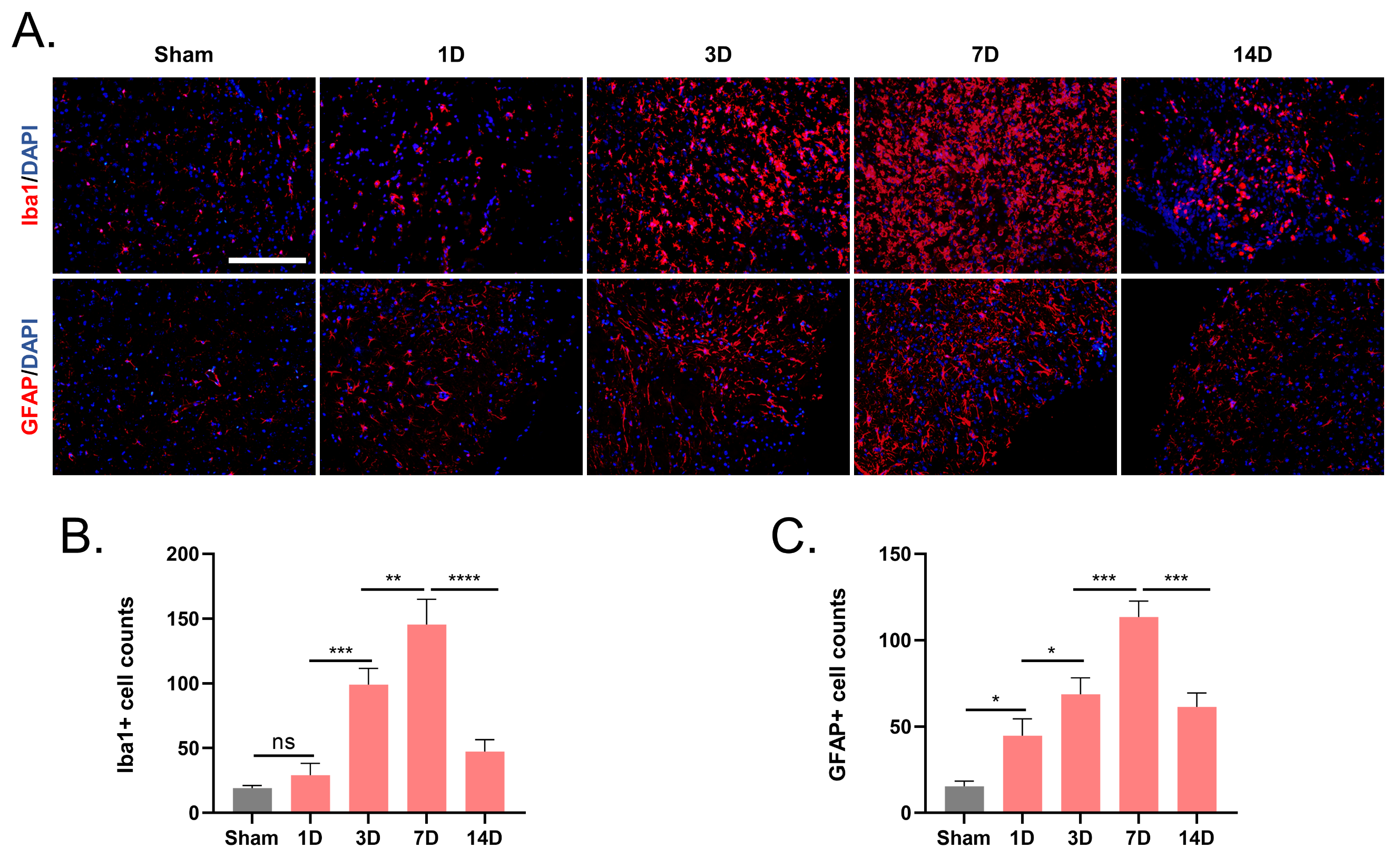


**Supplementary Figure 1. Exploration of the reactivity of microglia and astrocytes in the brain at different time points after TBI.** (A) Representative results of microglia and astrocyte immunofluorescence at different time points after TBI; (B) Quantitative statistics of Iba1+ cell counts in A; (C) Quantitative statistics of GFAP+ cell counts in A. n.s, P＞0.05; *, P≤0.05, **, P≤0.01; ***, P≤0.001; ****, P≤0.0001. The results are presented as mean ± S.D. n = 5/group


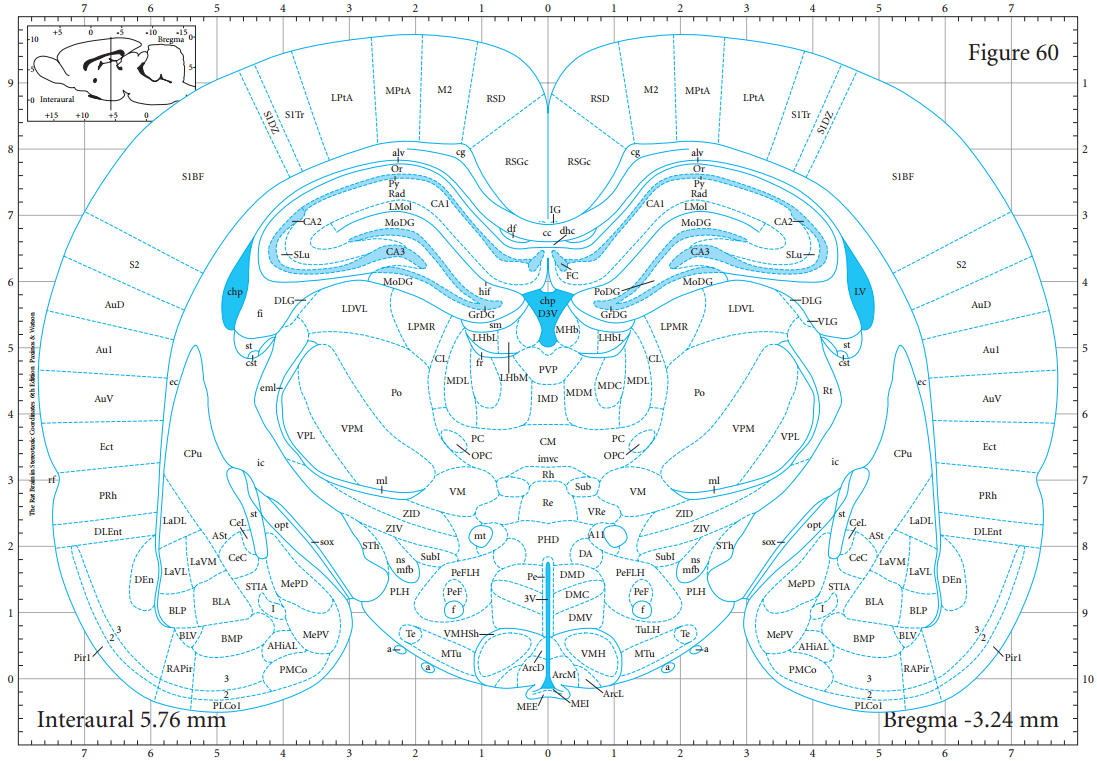


**Supplementary Figure 2. Rat brain atlas.** After hematoxylin and eosin (HE) staining, the hippocampal area of the ipsilateral cortex was visualized in conjunction with brain atlas.


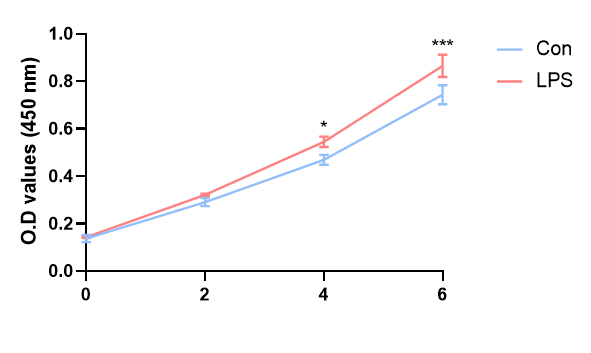


**Supplementary Figure 3.** Proliferation of microglia was measured by CCK8 method. Data are represented as mean ± SEM, n.s, P＞0.05; *, P≤0.05, **, P≤0.01; ***, P≤0.001; ****, P≤0.0001.


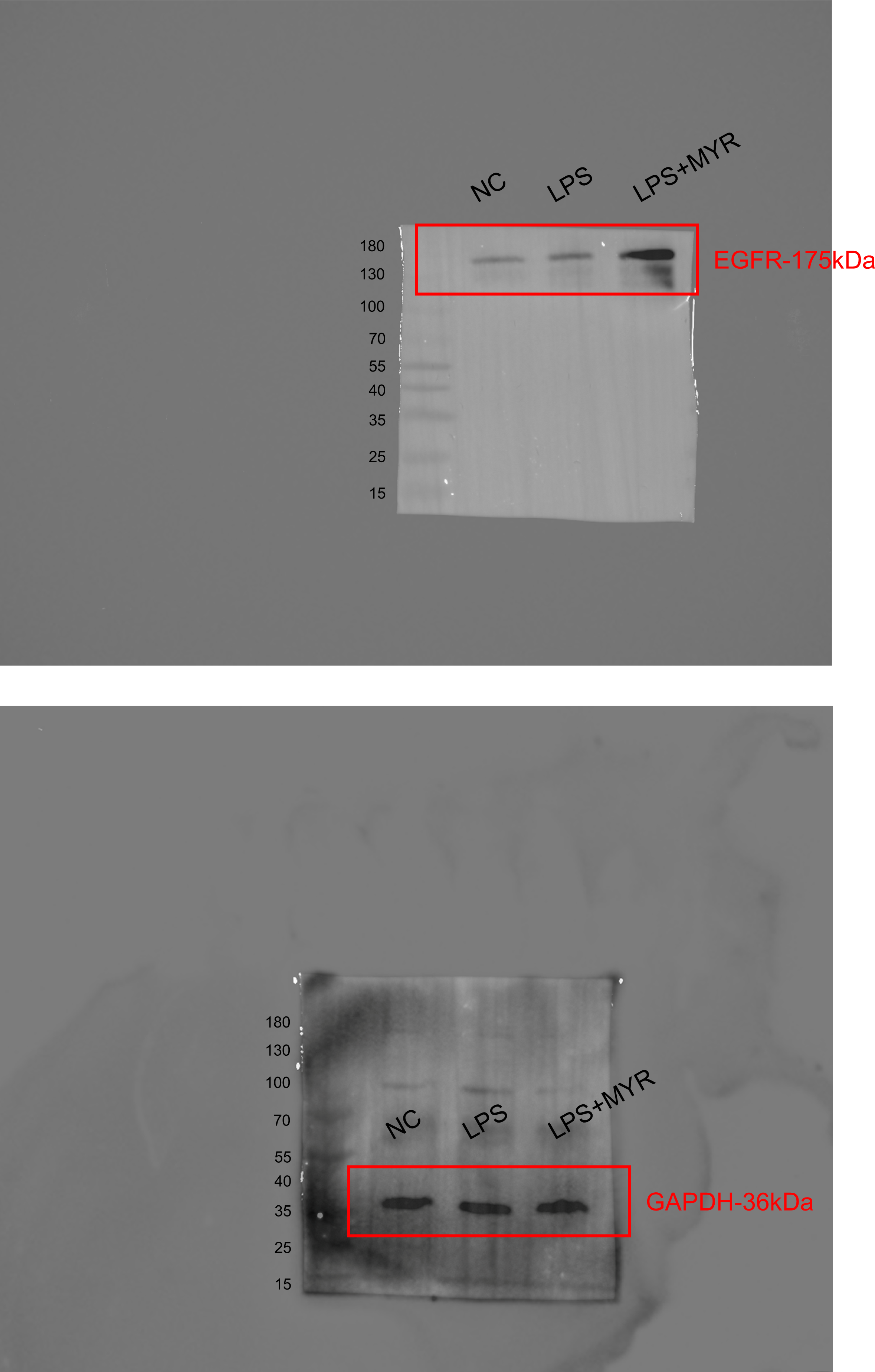


**Supplementary Figure 4. EGFR protein expression.**


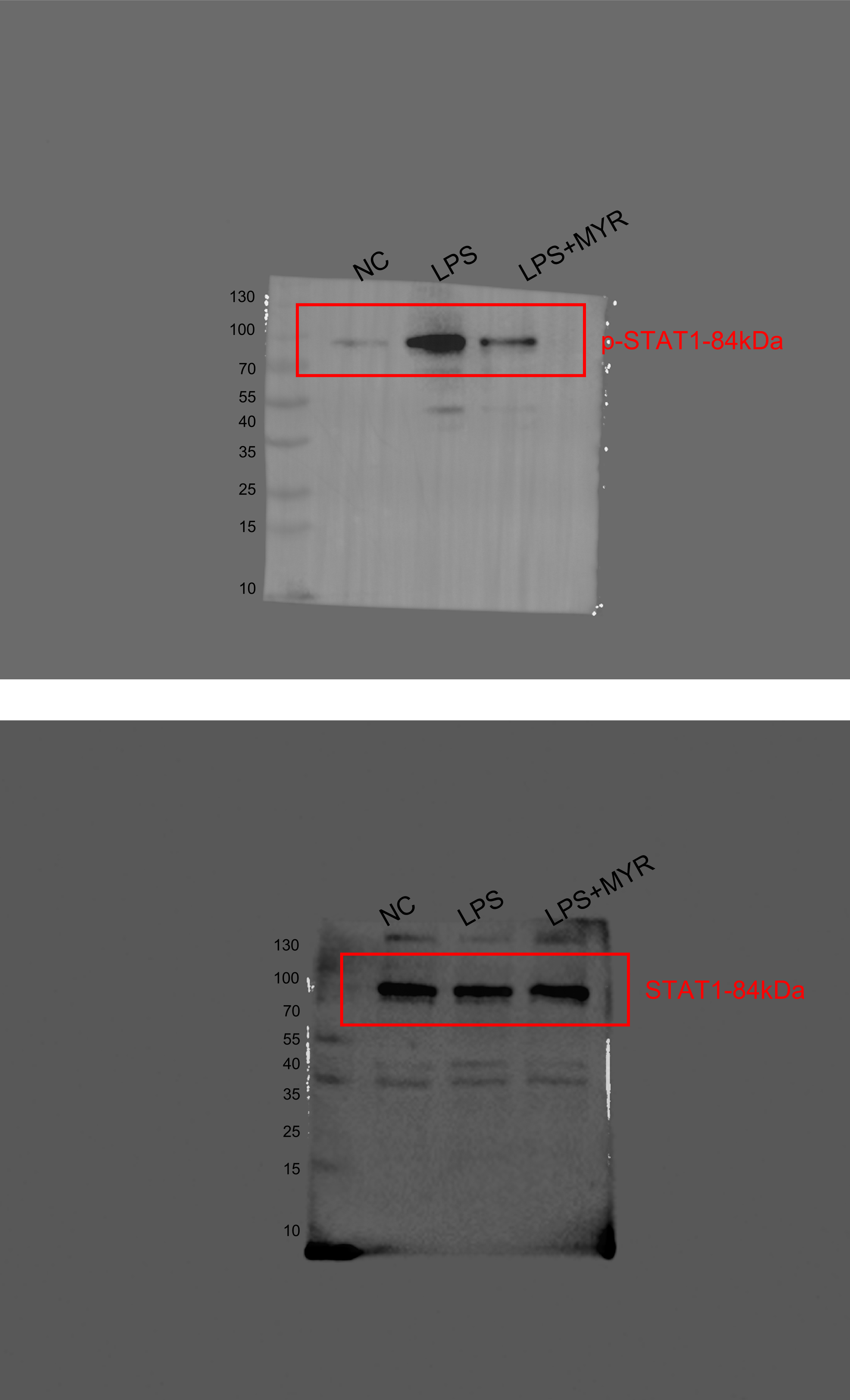


**Supplementary Figure 5. p-STAT1 protein expression.**


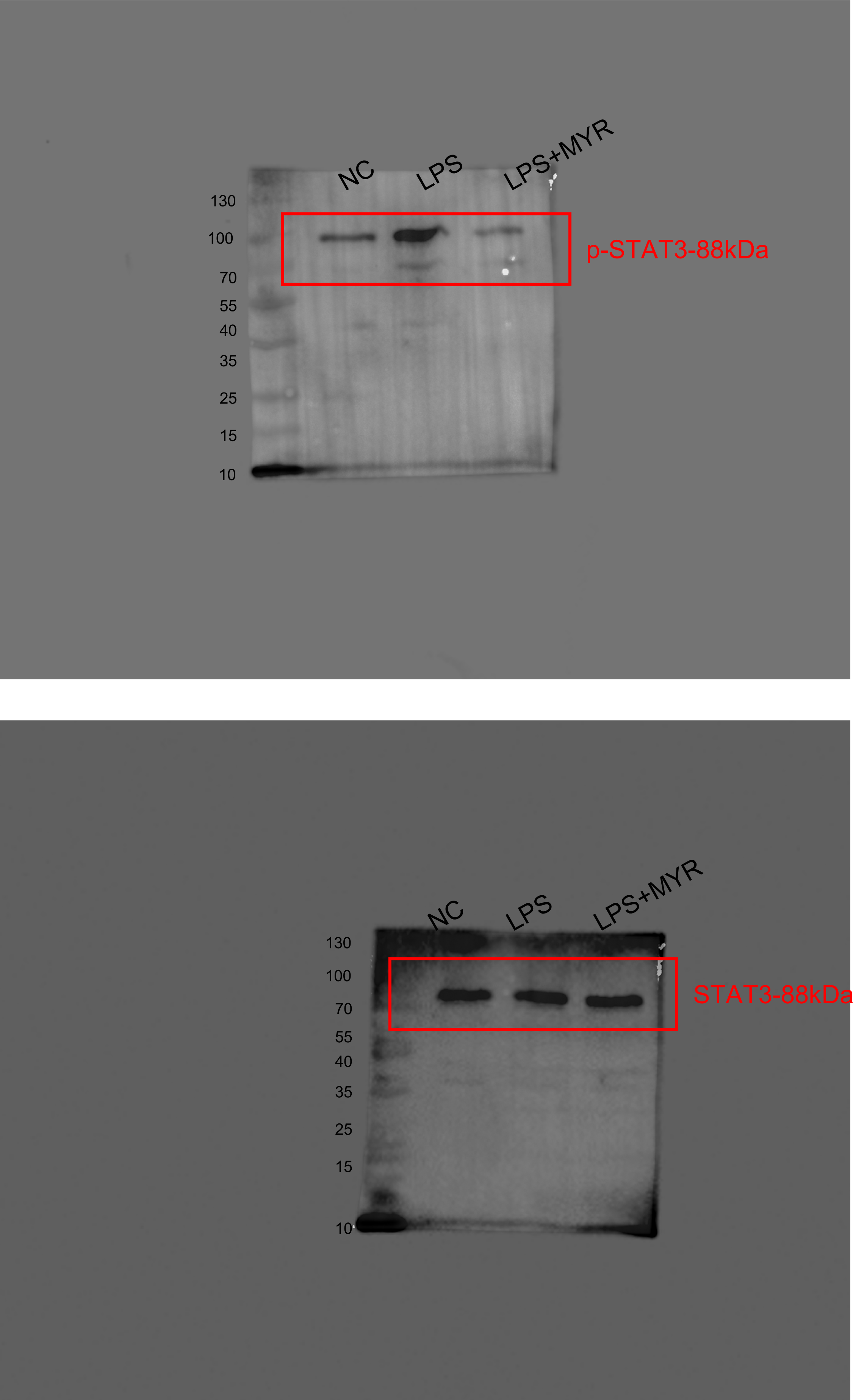


**Supplementary Figure 6. p-STAT3 protein expression.**


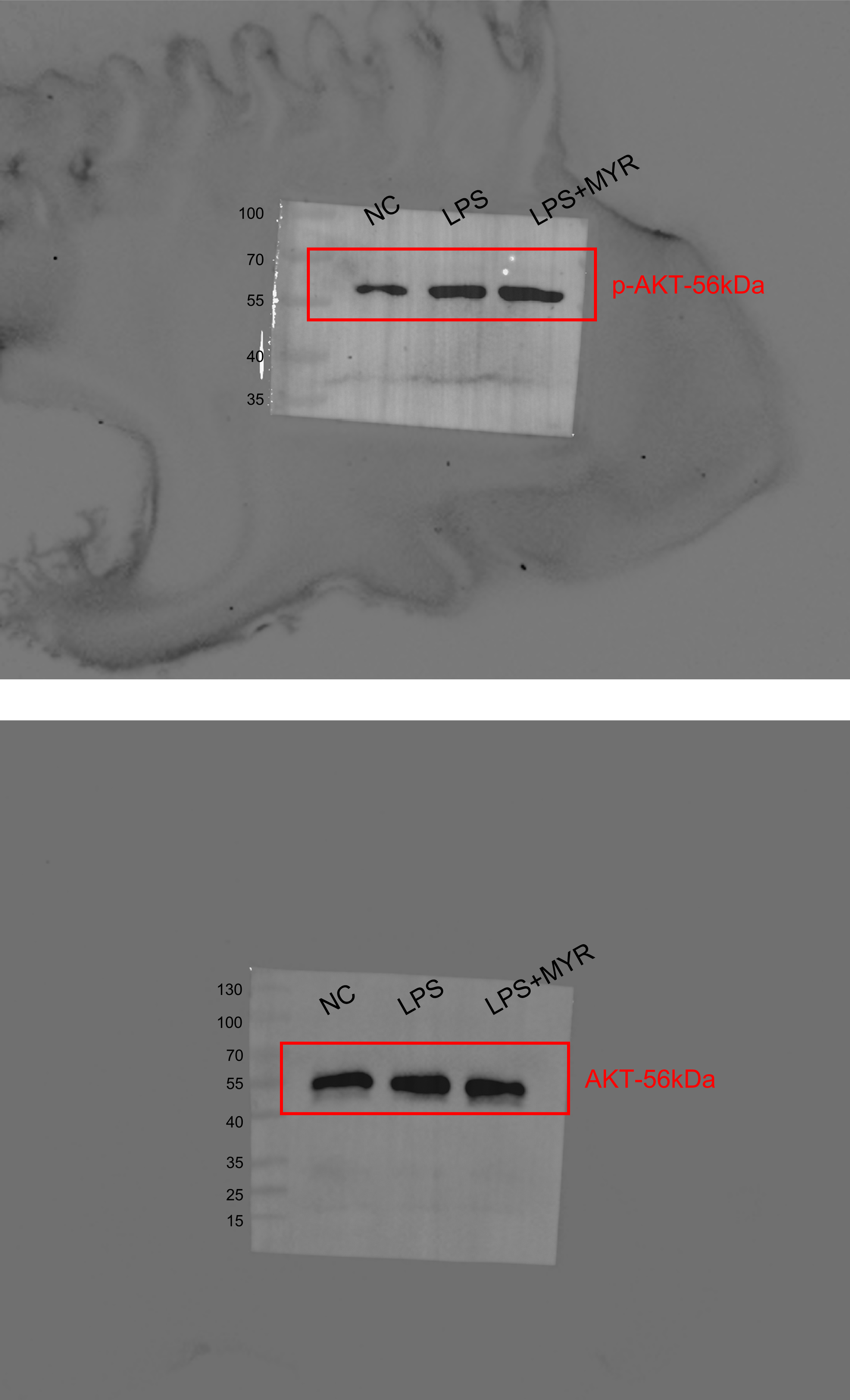


**Supplementary Figure 7. p-AKT protein expression.**
